# DCE-MRI of Tumor Hypoxia and Hypoxia-Associated Aggressiveness

**DOI:** 10.3390/cancers12071979

**Published:** 2020-07-20

**Authors:** Jon-Vidar Gaustad, Anette Hauge, Catherine S. Wegner, Trude G. Simonsen, Kjersti V. Lund, Lise Mari K. Hansem, Einar K. Rofstad

**Affiliations:** 1 Group of Radiation Biology and Tumor Physiology, Department of Radiation Biology, Institute for Cancer Research, Oslo University Hospital, 0310 Oslo, Norway; Anette.Hauge@rr-research.no (A.H.); Catherine.Sem.Wegner@rr-research.no (C.S.W.); Trude.Golimo.Simonsen@rr-research.no (T.G.S.); Kjersti.Vassmo@rr-research.no (K.V.L.); Lise.Mari.Klepp.Andersen@rr-research.no (L.M.K.H.); einar.k.rofstad@rr-research.no (E.K.R.); 2 Department of Radiology and Nuclear Medicine, Oslo University Hospital, 0310 Oslo, Norway

**Keywords:** tumor hypoxia, hypoxia-associated treatment response, hypoxia-induced metastasis, DCE-MRI, cervical carcinoma, melanoma, pancreatic ductal adenocarcinoma

## Abstract

Tumor hypoxia is associated with resistance to treatment, aggressive growth, metastatic dissemination, and poor clinical outcome in many cancer types. The potential of dynamic contrast-enhanced magnetic resonance imaging (DCE-MRI) to assess the extent of hypoxia in tumors has been investigated in several studies in our laboratory. Cervical carcinoma, melanoma, and pancreatic ductal adenocarcinoma (PDAC) xenografts have been used as models of human cancer, and the transfer rate constant (*K*^trans^) and the extravascular extracellular volume fraction (*v*_e_) have been derived from DCE-MRI data by using Tofts standard pharmacokinetic model and a population-based arterial input function. *K*^trans^ was found to reflect naturally occurring and treatment-induced hypoxia when hypoxia was caused by low blood perfusion, radiation responsiveness when radiation resistance was due to hypoxia, and metastatic potential when metastasis was hypoxia-induced. *K*^trans^ was also associated with outcome for patients with locally-advanced cervical carcinoma treated with cisplatin-based chemoradiotherapy. Together, the studies imply that DCE-MRI can provide valuable information on the hypoxic status of cervical carcinoma, melanoma, and PDAC. In this communication, we review and discuss the studies and provide some recommendations as to how DCE-MRI data can be analyzed and interpreted to assess tumor hypoxia.

## 1. Introduction

Regions with hypoxic tissue (pO_2_ < 10 mmHg) are a characteristic feature of most experimental and human tumors [1]. Preclinical studies have demonstrated that tumors with extensive hypoxia are resistant to several types of therapy and that tumor hypoxia can promote malignant progression and metastatic spread [1,2,3]. Clinical studies have shown that patients with highly hypoxic tumors have increased risk of treatment failure, increased frequency of metastases, and poor survival rates following radiation therapy, alone or in combination with surgery and chemotherapy [1]. If the hypoxic status of tumors could be assessed prior to treatment, patients with highly hypoxic tumors could be offered more aggressive or additional treatment to increase survival rates, whereas patients with well-oxygenated tumors could be offered reduced treatment doses to reduce side effects. Biomarkers reflecting the hypoxic status of tumors are thus highly warranted to individualize cancer treatment.

Positron emission tomography (PET) imaging using oxygen-sensitive radiotracers allows direct assessment of hypoxic tumor fractions [4]. Several oxygen-sensitive radiotracers have been used in clinical trials, including ^18^F-labeled fluoromisionidazole (FMISO), ^18^F-fluoroazomycin-arabinoside (FAZA), and ^60^Cu-diacetyl-bis(N4-methylthiosemicarbazone) (^60^Cu-ATSM), and the reports have been promising [5,6]. However, the spatial resolution associated with PET imaging is low compared to the size of the hypoxic regions that can be found in tumor tissue [5]. Magnetic resonance imaging (MRI) techniques, including blood oxygen level dependent MRI (BOLD-MRI), tissue oxygen level dependent MRI (TOLD-MRI), oxygen-enhanced MRI (OE-MRI), and dynamic contrast-enhanced MRI (DCE-MRI), have also been applied to study tumor hypoxia [4,5,7,8]. MRI can be performed with substantially higher spatial resolution than PET imaging, and DCE-MRI is highly attractive because the technique is associated with a high signal to noise ratio and is routinely used to detect and characterize various types of cancer in the clinic.

In DCE-MRI, a paramagnetic contrast agent (CA) is injected intravenously, and the CA uptake in organs or tumors is studied by recording multiple images within a few minutes. The uptake of CA in tumor tissue depends on several microenvironmental parameters, including blood perfusion, vessel wall permeability, cell density, extracellular volume fraction, and extracellular matrix density [9]. Tumor hypoxia is a result of an imbalance between oxygen supply and oxygen consumption [10]. The oxygen supply is primarily governed by the blood perfusion, whereas the oxygen consumption is mainly determined by the respiratory activity of the tissue and, hence, the cell density [11,12]. Tumor hypoxia is thus expected to be found in regions with low blood perfusion and/or high cell density, and DCE-MRI can potentially be used to identify these regions. Indeed, correlations have been found between DCE-MRI derived parameters and oxygen tension or the outcome of radiation therapy in some cancer types [13,14,15,16,17]. However, the correlations are generally weak, possibly because the DCE-MRI derived parameters used in these studies were inadequate measures of tumor hypoxia.

In our laboratory, we investigated whether DCE-MRI can be used to detect naturally occurring and treatment-induced tumor hypoxia, as well as to predict hypoxia-associated radiation response and hypoxia-induced metastasis, by using preclinical models of cervical carcinoma, melanoma and pancreatic ductal adenocarcinoma (PDAC). We also investigated whether DCE-MRI derived parameters can be used to predict outcome for patients with locally-advanced cervical carcinoma. In this communication, we review and discuss this work and provide recommendations as to how DCE-MRI can be used to detect tumor hypoxia.

## 2. Analysis of DCE-MRI Data

Calculation of CA concentration is recommended rather than using absolute or relative signal intensities in DCE-MRI data analysis [18]. This recommendation is made because signal intensities heavily depend on scanner settings and protocols, thus complicating comparison between different imaging facilities. In our laboratory, CA concentration has been calculated by using T_1_-maps [19] or proton density images [20] recorded before CA injection. Calculation of CA concentration by using T_1_-maps is relatively straight forward and is the preferred method for most instances, but the recording of accurate T_1_-maps typically requires 5–10 min scan time [18]. Proton density images can be recorded in 1–2 min, but phantoms with known CA concentration must be placed within the image frame, as described in detail by Hittmair et al. [21]. Placing phantoms within the image frame can easily be done for superficial tumors but can be impractical or impossible for tumors growing in deep organs.

**Figure 1:**
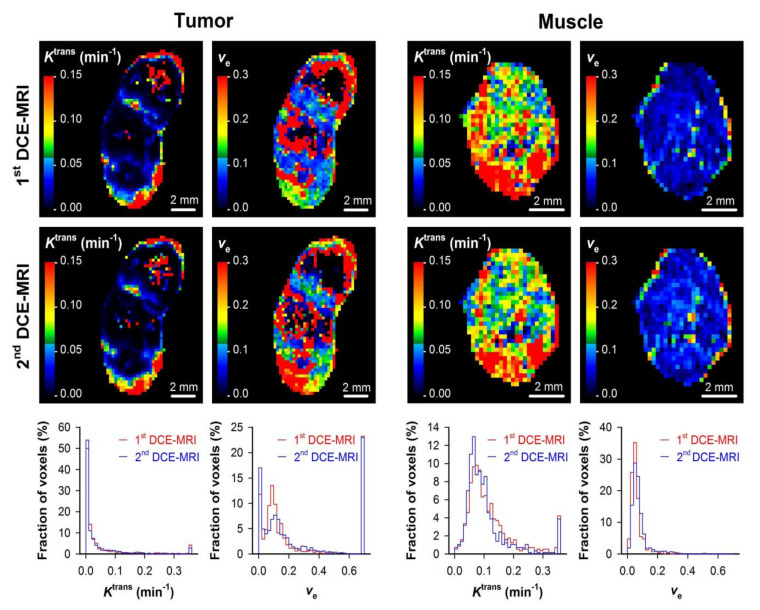
The reproducibility of the dynamic contrast-enhanced magnetic resonance imaging (DCE-MRI) acquisition and analysis was investigated by subjecting tumors and normal muscle tissue to DCE-MRI twice, with an imaging interval of 60 min. The figure shows the transfer rate constant (*K*^trans^) images, *K*^trans^ frequency distributions, extravascular extracellular volume fraction (*v*_e_) images, and *v*_e_ frequency distributions derived from the first and the second DCE-MRI session of a tumor and a tumor-free leg muscle and illustrates that highly reproducible data was obtained. Modified from Wegner et al. [24].

**Figure 2:**
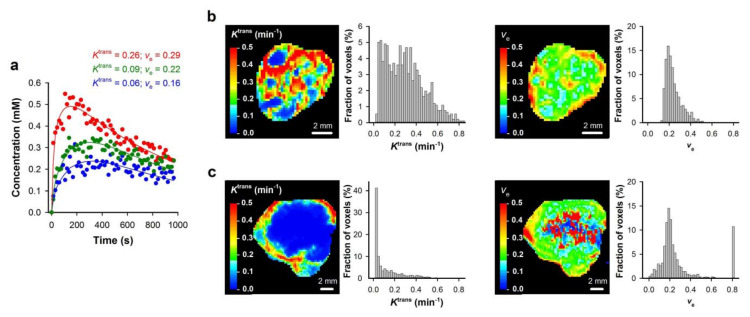
DCE-MRI series were analyzed on a voxel-by-voxel basis by using Tofts standard pharmacokinetic model and a population-based arterial input function. (**a**) Plots of gadolinium diethylene-triamine penta-acetic acid (Gd-DTPA) concentration versus time and the corresponding pharmacokinetic model fits for individual voxels. (**b**,**c**) *K*^trans^ image, *K*^trans^ frequency distribution, *v*_e_ image, and *v*_e_ frequency distribution of a TS-415 (**b**) and a CK-160 (**c**) cervical carcinoma xenograft. Modified from Ellingsen et al. [26].

Several pharmacokinetic models have been developed to analyze DCE-MRI data, including the Tofts standard pharmacokinetic model, the Brix model, and the shutter-speed model [9,22]. Among these, Tofts standard pharmacokinetic model has been reported to be preferable in analysis of human DCE-MRI data [22]. According to Tofts standard pharmacokinetic model, the CA concentration in tissue at time *T* is given by:

$$C_{t}\left(T\right)=\frac{K^{\mathrm{trans}}}{\left(1-\mathrm{Hct}\right)}\cdot \int_{0}^{T}C_{a}\left(t\right)\cdot e^{-\frac{K^{\mathrm{trans}}\cdot \left(T-t\right)}{v_{e}}}\;dt,$$

where *C*_a_(*t*) is the CA concentration in arterial blood, *K*^trans^ is the transfer rate constant, *v*_e_ is the extravascular extracellular volume fraction, and Hct is the hematocrit. We demonstrated that highly reproducible *K*^trans^ and *v*_e_ images can be produced by using Tofts standard pharmacokinetic model [23,24]. This is illustrated in Figure 1, which shows *K*^trans^ and *v*_e_ images of a tumor and normal muscle tissue subjected to DCE-MRI twice. Figure 2 shows *K*^trans^ and *v*_e_ images of cervical carcinoma xenografts, and CA concentration versus time plots and the corresponding pharmacokinetic model fits for individual voxels. The figure illustrates that good model fits can be obtained for voxels with both high and low uptake of CA. Similar time curves and model fits have been obtained for viable tissue in multiple cervical carcinoma, melanoma, and PDAC xenograft models [19,24,25,26]. We recommend that investigators document reproducibility and report CA concentration versus time plots and model fits for individual voxels to illustrate the quality of the DCE-MRI acquisition and analysis.

Tofts standard pharmacokinetic model is a two-compartment model, where the vasculature represents the first compartment, and the extravascular extracellular space represents the second compartment [9,27]. The model assumes that the CA is well mixed within each compartment. This assumption is not valid for necrotic and fibrotic tissue where the density of functional capillaries can be extremely low, and the diffusion distances in the extravascular extracellular space can be large [9,27,28]. We have shown that our algorithms produce unphysiological *v*_e_ values (often > 1000) for voxels consisting of necrotic or fibrotic tissue [28,29,30]. Most of these voxels also have low *K*^trans^ values, similar to voxels found in hypoxic tissue. It is important to exclude these voxels to avoid overestimation of the hypoxic fraction [20,30]. We obtained individual *v*_e_ threshold values for eight melanoma xenograft models by carefully comparing *v*_e_ images and histological preparations [20]. We also demonstrated that simply excluding voxels with *v*_e_ > 1.0 is sufficient to exclude the majority of unphysiological voxel values in cervical carcinoma and PDAC xenograft models [24,25]. We recommend using this general threshold value to exclude unphysiological voxels when histolological preparations of the imaged tissue are not available.

Knowledge of the CA concentration in arterial blood (the arterial input function, AIF) is required in most pharmacokinetic models, including Tofts standard pharmacokinetic model. If a large artery is present in the image frame, the AIF can (in principle) be measured from the DCE-MRI series. However, measurement of the AIF requires higher temporal resolution (typically 1–3 s) than measurements of CA concentration in tumor tissue (typically 10–20s). In our laboratory, we used a population-based AIF developed for mice rather than measuring individual AIFs [31]. The main advantage of using a population-based AIF is that imaging can be performed with improved spatial resolution and/or increased signal-to-noise ratio because the required temporal resolution is lower. A possible disadvantage is that the AIF may vary for individual mice, and any variation is ignored when using a population-based AIF. However, our population-based AIF was established by analyzing blood samples from 12 individual mice and the data points suggested that the AIFs did not differ between the mice [31]. We also developed individual AIFs by performing DCE-MRI of the heart of mice, and the individual AIFs did not differ from each other and did not differ from the population-based AIF [31]. These experiments suggest that using a population-based AIF does not represent a significant limitation when imaging genetically identical mice. One may suspect that AIFs in patients with different pathologies vary more than AIFs in genetically identical mice and that using individual AIFs for DCE-MRI analysis is necessary in a clinical setting. However, some investigators have argued that the uncertainties introduced by measuring individual AIFs may be comparable or even larger than the interpatient variation in AIF, and population-based AIFs have also been developed for patients [32]. We have shown that parameters derived by using a population-based AIF predict outcome for patients with locally-advanced cervical carcinoma [33].

## 3. *K*^trans^ Reflects Naturally Occurring Tumor Hypoxia

**Figure 3:**
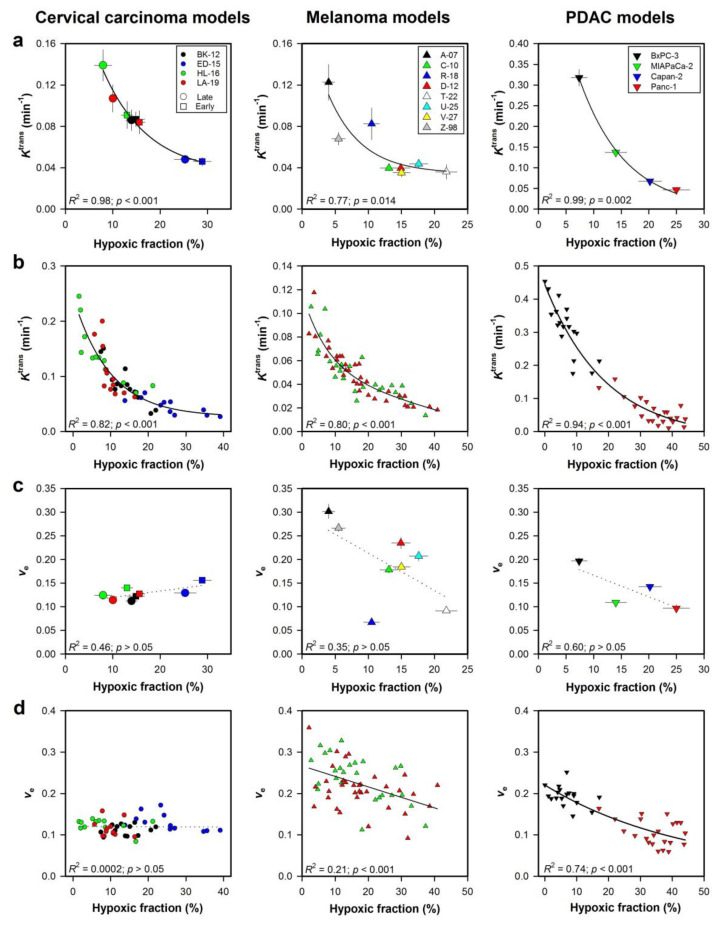
Xenografted tumors were subjected to DCE-MRI before the fraction of hypoxic tumor tissue was determined by immunohistochemistry. The figure shows *K*^trans^ versus hypoxic fraction (**a**,**b**), and *v*_e_ versus hypoxic fraction (**c**,**d**) in early and late passages of BK-12, ED-15, HL-16, and LA-19 cervical carcinoma xenografts, A-07, C-10, R-18, D-12, T-22, U-25, V-27, and Z-98 melanoma xenografts, and BxPC-3, MIAPaCa-2, Capan-2, and Panc-1 pancreatic ductal adenocarcinonma (PDAC) xenografts. Hypoxic fractions were measured in histological preparations by using pimonidazole as a hypoxia marker. Points represent mean of 9–28 tumors (**a**,**c**) or individual tumors (**b**,**d**). Bars represent SEM (**a**,**c**). Exponential and linear curves were fitted to the data by regression analysis. *p*-values were obtained by Pearson product moment correlation test. Modified from Hauge et al. [25], Egeland et al. [20], Øvrebø et al. [36], Wegner et al. [24], and Wegner et al. [37].

We investigated the potential of DCE-MRI to assess tumor hypoxia by using cervical carcinoma, melanoma, and PDAC xenografts as preclinical models of human tumors [20,24,25]. These tumor models differ considerably in histological appearance. The cervical carcinoma and PDAC xenografts both develop substantial stroma, but the connective tissue is more extensive, and the density of collagen fibers is higher in the PDAC than in the cervical carcinoma xenografts. In addition, in these models, many blood vessels are located within the connective tissue rather than in the tumor parenchyma, and this feature is more pronounced in the PDAC than in the cervical carcinoma xenografts. The melanoma xenografts show a sparse stroma with little collagen, and the majority of the blood vessels are not separated from the tumor parenchyma by connective tissue. Despite these differences, we found strong correlations between *K*^trans^ and hypoxic fraction for all the tumor models [20,24,25]. This is illustrated in Figure 3a, which shows plots of *K*^trans^ versus hypoxic fraction for 4 cervical carcinoma, 8 melanoma, and 4 PDAC xenograft models. Importantly, similar correlations between *K*^trans^ and hypoxic fraction were found both when mean values of the tumor models were considered (Figure 3a), as well as when values of individual tumors were examined (Figure 3b). Only weak or no correlations were found between *v*_e_ and hypoxic fraction (Figure 3c–d). These observations imply that *K*^trans^ reflected blood perfusion and oxygen supply, and imply that the heterogeneity in hypoxic fraction was caused by heterogeneity in oxygen supply rather than heterogeneity in cell density and oxygen consumption in these tumor models. The similar correlations between *K*^trans^ and hypoxic fraction in xenograft models with vast differences in extracellular matrix density and composition, imply that the extracellular matrix does not represent a substantial barrier to extravascular and interstitial transport of low-molecular weight CA, such as magnevist (Gd-DTPA) and dotarem (Gd-DOTA). 

A few clinical studies have compared DCE-MRI derived parameters with tumor oxygenation or the hypoxic tumor fraction in the same individual tumors. Loncaster et al. [15] measured tumor oxygenation by using the Eppendorf pO_2_ histograph in 35 cervical carcinoma patients and subjected the same tumors to DCE-MRI. They found correlations between tumor oxygenation and DCE-MRI derived parameters both when parameters were calculated from the increase in signal intensity without using pharmacokinetic models and when parameters were calculated by using the Brix pharmacokinetic model. The strongest correlations were found between tumor oxygenation and *A*_Brix_. *A*_Brix_ is the amplitude calculated by using the Brix pharmacokinetic model, and this parameter is strongly related to *K*^trans^ [9]. *A*_Brix_ was also correlated with a hypoxia gene signature in a study involving 80 cervical carcinoma patients [34]. In a small study involving 7 patients with head and neck cancer, correlations were found between tumor hypoxia assessed by pimonidazole-staining and perfusion parameters derived by DCE-MRI [35]. Although the clinical studies were rather small and different strategies were used to analyze and validate the DCE-MRI data, the studies support that DCE-MRI can provide information on the hypoxic status of tumors.

## 4. *K*^trans^ Reflects Radiation Responsiveness

**Figure 4:**
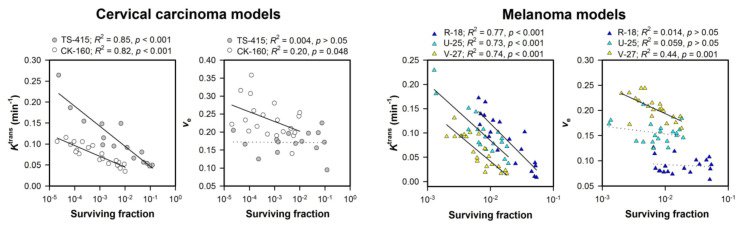
Xenografted tumors were subjected to DCE-MRI prior to fractionated or single-dose irradiation. The figure shows median *K*^trans^ versus cell surviving fraction and median *v*_e_ versus cell surviving fraction in TS-415 and CK-160 cervical carcinoma xenografts, and R-18, U-25, and V-27 melanoma xenografts. The cervical carcinoma xenografts were irradiated with 5 fractions of 4 gray (Gy) in 48 h, and the melanoma xenografts were irradiated with a single dose of 10 Gy. The cell survival of irradiated tumors was measured in vitro as detailed previously [39]. Briefly, the tumors were resected immediately after the last radiation fraction, minced in saline and treated with an enzyme solution. Trypan blue negative cells were plated in tissue culture flasks and incubated for 14 days for colony formation. The cell surviving fraction of an irradiated tumor was calculated from the number of cells plated, the number of colonies counted, and the mean plating efficiency of the cells. Points represent individual tumors, and curves were fitted to the data by linear regression analysis. *p*-values were obtained by Pearson product moment correlation test. Modified from Ellingsen et al. [26] and Øvrebø et al. [36].

It is well known that hypoxia causes radiation resistance [1]. To investigate whether *K*^trans^ and/or *v*_e_ values can predict radiation responsiveness, we subjected xenografted tumors to DCE-MRI before irradiation [26,36]. Strong correlations were found between *K*^trans^ and cell surviving fraction for cervical carcinoma and melanoma xenografts subjected to irradiation, whereas weak or no correlations were found between *v*_e_ and cell surviving fraction (Figure 4). In the same studies, strong correlations were also found between *K*^trans^ and the hypoxic tumor fraction, implying that *K*^trans^ reflected radiation responsiveness because *K*^trans^ was sensitive to hypoxia-induced radiation resistance [26,36]. Hallac et al. [38] subjected Dunning R3327-AT1 prostate tumors to DCE-MRI before irradiation and used tumor growth delay as parameter for radiation responsiveness. They found correlations between tumor growth delay and both *K*^trans^ and *v*_e_, suggesting that DCE-MRI is also sensitive to radiation responsiveness in models of prostate cancer.

## 5. *K*^trans^ Is Insensitive to Radiation-Induced Hypoxia

**Figure 5:**
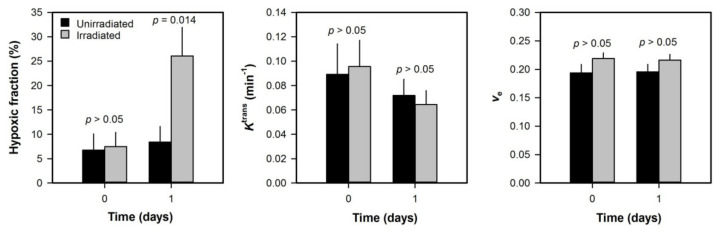
Xenografted tumors were subjected to DCE-MRI before and after irradiation. The figure shows hypoxic fraction, *K*^trans^, and *v*_e_ in unirradiated and irradiated A-07 melanoma xenografts before (day 0) and after (day 1) tumors were exposed to a single dose of 20 Gy. Hypoxic fractions were measured with a radiobiological assay. Columns represent mean of 6–10 tumors and bars represent SEM. *p*-values were obtained by Student’s *t*-test. Modified from Benjaminsen et al. [40].

The hypoxic tumor fraction may increase shortly after radiation exposure because aerobic cancer cells are inactivated, whereas the hypoxic cancer cells survive. To investigate whether *K*^trans^ and/or *v*_e_ are sensitive to radiation-induced hypoxia, we subjected melanoma xenografts to DCE-MRI before (day 0) and after (day 1) radiation exposure [40]. Although the hypoxic fraction increased after irradiation, no changes in *K*^trans^ nor *v*_e_ were observed (Figure 5). This observation illustrates that *K*^trans^ and *v*_e_ are insensitive to increases in hypoxic fractions that are not caused by reduced blood perfusion or increased cell density. This important limitation applies because DCE-MRI does not measure oxygenation directly but rather provides information on the oxygen supply and oxygen consumption. If the hypoxic fraction is increased without changes in the oxygen supply or oxygen consumption, DCE-MRI cannot be expected to reflect the increase in hypoxic fraction.

## 6. *K*^trans^ Reflects Hypoxia Induced by Antiangiogenic Treatment

**Figure 6:**
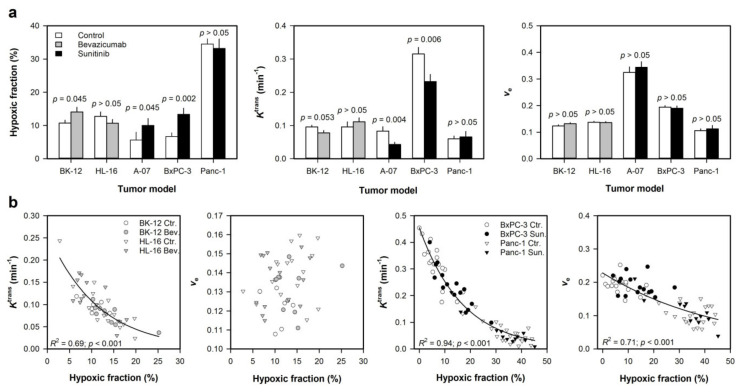
Tumors were subjected to DCE-MRI and measurement of hypoxic fraction after antiangiogenic treatment. Tumor-bearing mice were given 3 doses of 10 mg/kg bevacizumab over a period of 8 days (BK-12 and HL-16 cervical carcinoma xenografts), or daily doses of 40 mg/kg sunitinib for 4 days (A-07 melanoma and BxPC-3 and Panc-1 PDAC xenografts). (**a**) Hypoxic fraction, *K*^trans^, and *v*_e_ in untreated (control) and treated tumors. (**b**) Median *K*^trans^ versus hypoxic fraction and median *v*_e_ versus hypoxic fraction in untreated and treated tumors. Hypoxic fractions were measured in histological preparations by using pimonidazole as a hypoxia marker. Columns represent mean of 9–22 tumors and bars represent SEM (**a**). Points represent individual tumors, and exponential curves were fitted to the data by regression analysis (**b**). *p*-values were obtained by Student’s *t*-test (**a**) or Pearson product moment correlation test (**b**). Modified from Hauge et al. [42], Gaustad et al. [43], and Wegner et al. [37].

Several strategies have been developed to inhibit angiogenesis [41]. The antiangiogenic drugs reduce vessel density and can thus lower tumor perfusion and induce tumor hypoxia. In our laboratory, we investigated the effect of bevacizumab, a humanized antibody against vascular endothelial growth factor A (VEGF-A), and sunitinib, a tyrosine kinase inhibitor targeting several receptors, including VEGF receptors 1–3 [37,42,43]. The antiangiogenic drugs increased the hypoxic fraction in some but not all cervical carcinoma, melanoma, and PDAC xenograft models (Figure 6a). The treatment-induced hypoxia was reflected by reduced *K*^trans^ values, whereas the tumor models that did not respond did not show altered *K*^trans^ values (Figure 6a). Importantly, strong correlations were found between *K*^trans^ and the hypoxic fraction of individual tumors, and the correlations were similar for untreated and treated tumors (Figure 6b). Furthermore, treated and untreated tumors showed similar *v*_e_ values, and only weak or no correlations were found between *v*_e_ and the hypoxic fraction of individual tumors. Taken together, these observations imply that *K*^trans^ but not *v*_e_ is sensitive to hypoxia induced by antiangiogenic treatment, probably because the treatment increased the hypoxic fraction by reducing blood perfusion and did not alter cell density. Our experiments correspond well with a study including 13 cervical carcinoma patients treated with sorafinib, a tyrosine kinase inhibitor targeting VEGF receptors [44]. Sorafinib treatment induced hypoxia as revealed by the Eppendorf pO_2_ histograph and staining of the endogenous hypoxia markers hypoxia inducible factor 1 (HIF-1) and carbonic anhydrase IX (CAIX). Importantly, the sorafinib-treated tumors also showed reduced *K*^trans^.

## 7. *K*^trans^ Reflects Metastatic Potential

**Figure 7:**
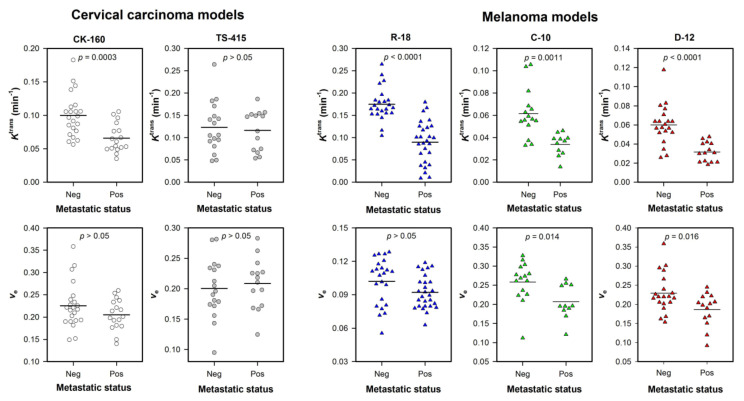
Tumors were subjected to DCE-MRI before the tumor-bearing mice were euthanized and examined for lymph node and pulmonary metastasis. The figure shows median *K*^trans^ and median *v*_e_ in metastasis-negative and metastasis-positive CK-160 and TS-415 cervical carcinoma xenografts, and R-18, C-10, and D-12 melanoma xenografts. Mice with CK-160, TS-415, and R-18 tumors showed lymph node metastasis, and C-10 and D-12 tumors developed spontaneous pulmonary metastasis. Points represent individual tumors and lines represent mean values. *p*-values were obtained by Student’s *t*-test. Modified from Ellingsen et al. [26], Øvrebø et al. [36], and Øvrebø et al. [45].

The cervical carcinoma and melanoma xenograft models established in our laboratory show different metastatic patterns. Some models metastasize to lymph nodes, and others develop spontaneous lung metastases [26,36,45]. To investigate whether *K*^trans^ and/or *v*_e_ values were sensitive to the metastatic potential, primary tumors were subjected to DCE-MRI before the tumor-bearing mice were euthanized and examined for lymph node and pulmonary metastasis. Tumors that developed lymph node or pulmonary metastases had lower *K*^trans^ values than tumors that did not develop metastases in all the investigated xenograft models except the TS-415 model (Figure 7). Interestingly, correlations have been found between tumor hypoxia and spontaneous metastasis in all the models except the TS-415 model, where elevated interstitial fluid pressure (and not hypoxia) has been identified as an important promoter of metastatic dissemination [26,36,45]. These observations imply that *K*^trans^ can reflect metastatic potential when metastasis is hypoxia-induced but not necessarily when metastasis is induced by other mechanisms. Only small or no differences in *v*_e_ were found between metastasis-positive and metastasis-negative tumors (Figure 7). Studies comparing DCE-MRI derived parameters with the metastatic potential of individual tumors are sparse. However, in a study involving 13 patients with ocular melanoma, Wei et al. [46] found that metastatic tumors had lower *K*^trans^ than non-metastatic tumors, in line with our experiments.

## 8. *K*^trans^ Predicts Outcome in Patients with Cervical Carcinoma

**Figure 8:**
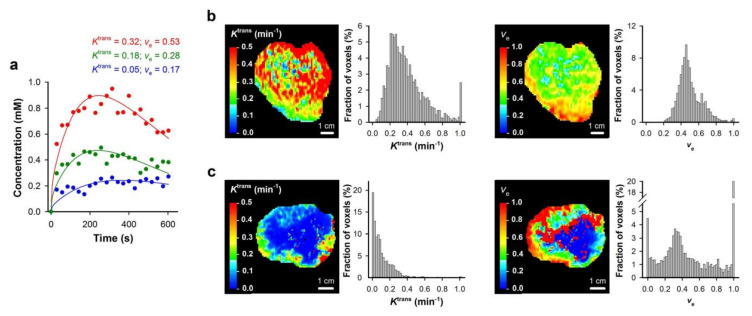
DCE-MRI series of human tumors were analyzed on a voxel-by-voxel basis by using Tofts standard pharmacokinetic model and a population-based arterial input function. (**a**) Plots of Gd-DTPA concentration versus time and the corresponding pharmacokinetic model fits for individual voxels. (**b**,**c**) *K*^trans^ image, *K*^trans^ frequency distribution, *v*_e_ image, and *v*_e_ frequency distribution of locally-advanced cervical carcinoma in two representative patients. Modified from Lund et al. [33].

**Figure 9:**
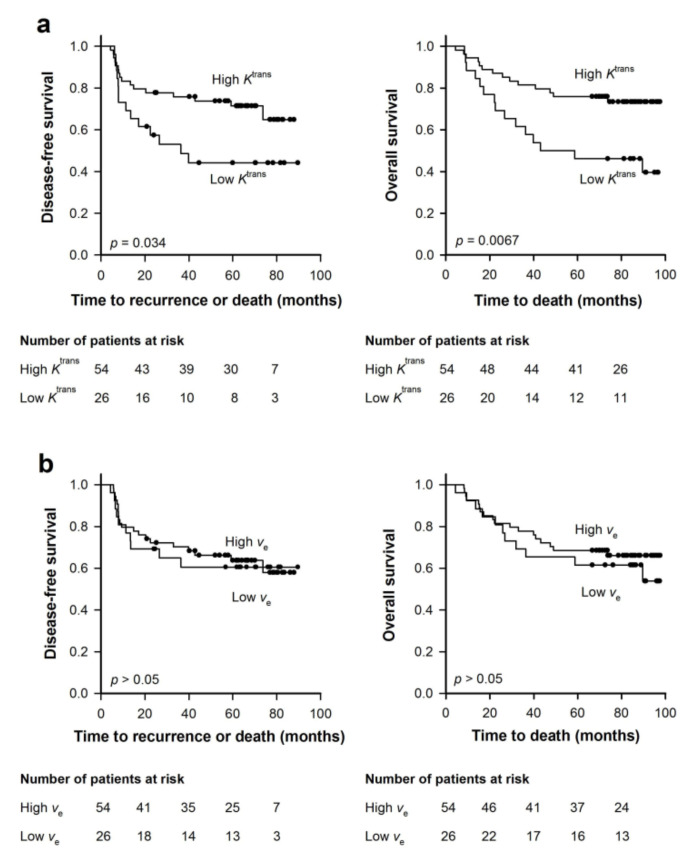
Eighty patients with locally-advanced cervical carcinoma were subjected to DCE-MRI prior to cisplatin-based chemoradiotherapy. The patients were divided in two groups consisting of one-third and two-thirds of the patients. The figure shows Kaplan–Meier curves for disease-free and overall survival of the 26 patients with lowest and the 54 patients with highest median *K*^trans^ (**a**), and the 26 patients with lowest and the 54 patients with highest median *v*_e_ (**b**). *p*-values were obtained by log-rank test. Modified from Lund et al. [33].

Tumor hypoxia is a major cause of treatment failure in cervical carcinoma patients given cisplatin-based chemoradiotherapy [47,48]. To investigate whether *K*^trans^ and/or *v*_e_ can predict outcome for this patient group, 80 patients with locally-advanced cervical carcinoma were subjected to DCE-MRI prior to cisplatin-based chemoradiotherapy [33]. Figure 8 shows *K*^trans^ and *v*_e_ images of two patients, as well as plots of CA concentration versus time and the corresponding pharmacokinetic model fits for individual voxels. The figure illustrates that the quality of the DCE-MRI data obtained in human tumors was sufficient to distinguish individual voxels with different CA uptake and to produce good model fits. The patients were divided in two groups consisting of patients with high *K*^trans^ (two third of the patients) and patients with low *K*^trans^ (one third of the patients). Equivalent groups were also made based on *v*_e_. This grouping is in accordance with the overall observation that standard first-line treatment fails in approximately one-third of patients with locally-advanced cervical carcinoma [49]. Figure 9 shows Kaplan–Meier plots for disease-free and overall survival for patients with high and low *K*^trans^ (Figure 9a) and high and low *v*_e_ (Figure 9b). The patients with high *K*^trans^ showed higher disease-free and overall survival rates than the patients with low *K*^trans^, whereas the survival rates of patients with high *v*_e_ did not differ from the patients with low *v*_e_.

DCE-MRI parameters derived by using Brix pharmacokinetic model and non-model based parameters calculated from the increase in signal intensities have also been shown to predict outcome in patients with cervical carcinoma, both by our group and others [15,34,50,51]. Moreover we have shown that risk volumes defined by using non-model based parameters (i.e., the low enhancing tumor volume) are strongly correlated to risk volumes defined by using *A*_Brix_ [50] or *K*^trans^ [33]. These observations suggest that prognostic biomarkers can be derived from DCE-MRI data by using non-model-based calculations, the Brix pharmacokinetic model, as well as the Tofts standard pharmacokinetic model. However, as reviewed here, the parameters derived by Tofts standard pharmacokinetic model reflect tumor physiology in a broad specter of tumor models, and Tofts model has been reported to be preferable in analysis of human DCE-MRI data [22].

## 9. Conclusions and Recommendations

We have demonstrated that *K*^trans^ derived from DCE-MRI data by using Tofts standard pharmacokinetic model and a population-based AIF is associated with tumor hypoxia in xenograft models of cervical carcinoma, melanoma, and PDAC. *K*^trans^ reflected naturally occurring and treatment-induced hypoxia when hypoxia was caused by low blood perfusion, radiation responsiveness when radiation resistance was due to hypoxia, and metastatic potential when metastasis was hypoxia-induced. However, *K*^trans^ did not reflect treatment-induced hypoxia when the treatment specifically inactivated aerobic cells (i.e., increased hypoxic fraction without reducing blood perfusion) and did not reflect metastatic potential when metastasis was induced by elevated interstitial fluid pressure (and not hypoxia). The similar correlations between *K*^trans^ and hypoxic fraction observed for xenograft models differing substantially in extracellular matrix density and composition strongly suggest that the extracellular matrix does not represent a substantial barrier for extravascular and interstitial transport of low-molecular weight CA. *K*^trans^ and *v*_e_ images were produced also for patients with locally-advanced cervical carcinoma, and *K*^trans^ was associated with survival after cisplatin-based chemoradiotherapy in this patient group. Tofts standard pharmacokinetic model is not valid for fibrotic and necrotic tissue, and voxels consisting mainly of fibrotic and necrotic tissue should be excluded to avoid overestimation of hypoxic fractions. We recommend that voxels with *v*_e_ > 1.0 are excluded when histological preparations of the imaged tissue are not available.
